# Evaluation of clopidogrel response in healthy cats using a novel viscoelastic test and thromboelastography

**DOI:** 10.3389/fvets.2024.1371781

**Published:** 2024-06-18

**Authors:** Tommaso Rosati, Karl E. Jandrey, Joshua A. Stern, Nghi Nguyen, Ronald H. L. Li

**Affiliations:** ^1^WR Pritchard Veterinary Medical Teaching Hospital, University of California Davis, Davis, CA, United States; ^2^Department for Small Animals, Vetsuisse Faculty, University of Zurich, Zurich, Switzerland; ^3^Department of Surgical and Radiological Sciences, University of California Davis, Davis, CA, United States; ^4^Department of Clinical Sciences, College of Veterinary Medicine, North Carolina State University, Raleigh, NC, United States; ^5^Department of Medicine and Epidemiology, University of California Davis, Davis, CA, United States

**Keywords:** clopidogrel resistance, arterial thromboembolic event (ATE), monitoring, light-transmission aggregometry, hypertrophic cardiomyopathy

## Abstract

**Introduction:**

Cats with cardiomyopathy face an increased risk of arterial thromboembolism (ATE). Although clopidogrel is frequently utilized to mitigate this risk, feline responses to this therapy exhibit variability. This study evaluated 2 viscoelastic devices, thromboelastography (TEG) and Viscoelastic Coagulation Monitor (VCM), for monitoring clopidogrel in cats in comparison to light transmission aggregometry (LTA).

**Methods:**

Twenty-eight healthy cats received clopidogrel for 7 days. Blood was collected at baseline and after treatment for analysis by TEG, VCM, and LTA.

**Results:**

On LTA, maximum amplitude, slope, and area under the curve (AUC) significantly decreased after treatment (*p* < 0.0001). On VCM, maximum clot firmness (MCF) significantly increased after treatment (*p* = 0.002). On TEG, R-time significantly prolonged (*p* = 0.024), while K and alpha angle significantly changed (*p* = 0.0002 and *p* = 0.0014, respectively). There was a moderate negative correlation between TEG R-time and LTA AUC (*r* = −0.39, *p* = 0.042). Eight cats were identified as non-responders to clopidogrel. Of the 8 non-responders, 6 (75%) had shortened R time after treatment. VCM appeared to be less discriminatory in identifying non-responders.

**Discussion:**

LTA remained the gold standard of monitoring clopidogrel treatment in cats. Unexpected changes on VCM and TEG were likely related to high interindividual and assay variability and increased sensitivity of feline platelets. R-time on TEG may have potential utility for point-of-care monitoring of clopidogrel response in cats.

## 1 Introduction

Cats with cardiomyopathies have increased risk to develop arterial thromboembolism (ATE). The reported survival rate for ATE in the feline population ranges from 35 to 50% (1). Platelets contribute to the pathogenesis of ATE in cats affected by hypertrophic cardiomyopathy (HCM) through an increased expression of P-selectin, a biomarker of platelet activation (2). Clopidogrel is an antiplatelet drug that irreversibly inhibits P2Y_12_, a platelet ADP receptor, and is commonly prescribed in HCM cats to reduce the risk of intracardiac thrombosis and ATE. Although clopidogrel has been shown to be superior to aspirin in reducing ATE recurrence in cats that survived a primary ATE event, recurrence remains, which is likely caused by the highly variable response to clopidogrel in cats (3–5).

Similarly, in human medicine, great emphasis is placed on platelet function monitoring in patients at high risk of recurrent thrombotic events due to a high variability in the pharmacodynamic effect of clopidogrel among individuals (6, 7). In humans, this is largely due to polymorphisms in the *CYP2C19* gene, responsible for encoding a member of the cytochrome P450 enzyme that mediates the biotransformation of the pro-drug into the active metabolite (8). A study by Ueda et al. demonstrated that clopidogrel resistance in a population of cats with HCM was due to a single nucleotide polymorphism in the *P2RY1* gene, which encodes one of the ADP receptors, P2Y_1_. Cats with this variant consistently showed reduced response to clopidogrel measured by ADP-induced platelet aggregation (5). In addition, various *ex vivo* studies have shown variable response and possible resistance to clopidogrel in up to 15 to 35% of cats. Although no direct associations between clopidogrel resistance and ATE has been found in cats, it is well documented in human medicine that resistance to the drug is associated with recurrent thrombotic events after myocardial infarction (6, 7). This emphasizes the importance of optimizing antiplatelet therapy on an individualized approach. One way to overcome the variable pharmacodynamics of clopidogrel is to monitor on-treatment platelet function. In human medicine, many different tests are available to evaluate platelet function in patients receiving antiplatelet drugs (8, 9). Although light transmission or whole blood impedance aggregometry are considered the gold standard technology, these assays require dedicated equipment and specialized training to operate making their use in general practice impractical (9). In humans, several studies investigated the sensitivity of viscoelastic testing to assess platelet function such as platelet mapping with conflicting results (10). In veterinary medicine, a few diagnostic tests such as flow cytometry have been investigated for monitoring of clopidogrel induced platelet inhibition (11, 12). Unfortunately, due to the limited distribution outside of advanced laboratories and the requirement of trained personnel, platelet function monitoring remains an option exclusive for academic settings and advanced medical centers.

Viscoelastic Platelet Mapping on TEG, which utilizes separate assessments of clot formation due to thrombin, fibrin and platelet function, have been studied in dogs and humans to assess pharmacodynamic effects of antiplatelet therapy. However, increased platelet sensitization and inter assay variation in cats likely limit its ability to detect antiplatelet effects (13). VCM Vet (Entegrion, Inc.) is a novel portable, compact, and user-friendly viscoelastic diagnostic device. A small amount of whole blood without anticoagulant or activators can be processed at the point-of-care to assess global coagulation (14). The VCM has been validated in a healthy feline population, as well as, in various other animal species (15). Owing to the species differences in platelet physiology and the hyper-responsive nature of feline platelets, TEG or VCM may be able to assess changes in platelet function without any specialized equipment, agonists or modifications (15). Hence the VCM may be a viable option to monitor clopidogrel therapy in cats in a clinical setting.

The primary aim of this study was to compare the performance of the novel device, VCM Vet, to kaolin-activated thromboelastography (TEG), and light-transmission aggregometry (LTA), the gold standard, in monitoring clopidogrel treatment and detecting non-responders to clopidogrel in cats. Our secondary aim was to correlate changes between VCM Vet, TEG and the gold standard, LTA.

## 2 Materials and methods

This prospective study was reviewed and approved by the University of California, Davis Institutional Animal Care and Use Committee (IACUC #20359).

Thirty healthy cats from a pathogen-free, university-owned colony of domestic shorthaired cats were enrolled in this study. Given a two-tailed design with an alpha level of 0.05 and an effect size of 15%, we expected to identify significant differences with an 80% power with a sample size of 22 cats. This calculation was based on standard deviation of the change in ADP-induced platelet aggregation in a previous study investigating the effect of clopidogrel in healthy cats by LTA (4). Given the likelihood of difficulties in sample collection and possible clotting of blood samples, additional enrollment of eight cats was allowed and the total number of cats enrolled was increased to 30. All cats were identified as healthy based on general physical examination, CBC and biochemistry performed before the study. None of the animals were on any medications for the duration of the study. All cats in the colony were housed in groups and had access to enrichment activities like daily social interactions with humans, toys, boxes and shelves. Drug administration and blood draw were timed so that blood collection could be performed only in 4 cats per day (2 in the morning and 2 in the afternoon). Blood collection was performed 5 to 15 min apart to minimize the variation of duration between sampling and assays.

### 2.1 Blood sampling

All blood samples were obtained without sedation. Blood was drawn using a 21 Gage needle from the jugular vein or 22 Gage butterfly needle from the medial saphenous vein. Approximately 6 mL of blood was drawn from each animal on the day of the experiment. Whole blood was separated into aliquots of three vials of citrated blood containing 3.2% sodium citrate and 1 vial of nonanticoagulated blood. Citrated samples were placed on a rack in an insulated container at room temperature and delivered to the central laboratory within 30 min of collection.

The remaining nonanticoagulated whole blood (300 μL) in polypropylene syringes was placed immediately into a pre-warmed testing cartridge before insertion into the point-of-care viscoelastic test (VCM Vet, Durham, NC). One citrated tube was used for CBC analysis (HM5, Abaxis, Parsippany, NJ) and platelet count was adjusted due to the 10% dilution effect of citrate.

### 2.2 Thromboelastography

Thromboelastography was performed on citrated whole blood within 2 h of collection. TEG sample processing (ie, waiting time, temperature) was conducted according to our clinical laboratory standardized protocol. Upon arrival at the laboratory, samples were maintained at room temperature and gently inverted 3 to 5 times immediately prior to performing the test. After 1 mL of citrated blood was mixed with kaolin (cat. no. 6300, Haemonetics Corp, Boston, MA), an aliquot of 360 ul of kaolin-activated blood was added to pre-warmed plain cup with 20 μL of CaCl_2_ and analyzed (TEG 5000, Haemonetics Corp, Boston, MA). The analysis was interrupted after running the samples for 60 min.

### 2.3 Generation of platelet rich plasma

Citrated blood was transferred to round-bottom polypropylene tubes and placed in 37^°^ C bead bath for 30 min to facilitate sedimentation of erythrocytes. Platelet rich plasma (PRP) was then generated by centrifugation at 200 × g for 5 min (no brakes, 25–27° C). Complete blood count of PRP was performed using an automated analyzer (HM5 Hematology analyzer, Zoetis, Parsippany, NJ) and confirmed by blood smear evaluation, within 2 h after collection. Platelet count was adjusted to account for the 10% dilutional effect of citrate.

### 2.4 Light transmission aggregometry

Platelet rich plasma generated from citrated whole blood was diluted to 1.5 × 10^8^ /mL using Tyrodes-HEPES (pH 7.2, 5 mM dextrose, no divalent cations) as described^4^. Platelet poor plasma, generated by centrifugation at 10,000 × g for 5 min, from each cat served as the control light transmission. PRP was aliquoted to prewarmed siliconized cuvettes (Chronolog, Havertown, PA) containing a magnetic stir bar set at a constant stir speed of 1,200 rpm. Aggregation was then recorded for 1 min as baseline before the addition of 40 μM ADP or 1 U/mL bovine α-thrombin (Haematologic Technologies, Essex Junction, VT), utilized as positive control. Aggregation was recorded for an additional 5 min and measured as percent (%) of maximum aggregation, slope, and area under the curve (AUC) transmission using commercially available software (Chronolog, Havertown, PA). The remaining plasma was used for biochemistry analysis (total protein, albumin, blood urea nitrogen, and creatinine) using automated biochemical analyzer (Vetscan 2, Abaxis, Parsippany, NJ).

### 2.5 Drug administration

After baseline measurements (Day 0), each cat received 18.75 mg clopidogrel bisulfate (Plavix, Britol-Myers Squibb/Sanofi Pharmaceuticals, Bridgewater, NJ) every 24 h by mouth for seven consecutive days. The tablet was administered using a pilling device, and the administration was confirmed by observing the cat swallowing. During the study period, all cats were monitored closely for adverse effects such as vomiting, lethargy and bleeding diathesis.

On Day 8, approximately 18 h after the last dose of clopidogrel, another 6 mL of blood was drawn for each cat. The obtained blood was separated into aliquots identical to Day 0 and was utilized to repeat CBC, VCM Vet, TEG and LTA as described above.

### 2.6 Response to clopidogrel

For each analyte obtained from each tested device VCM Vet (CT, clot time; CFT, clot formation time; AA, alpha-angle; MCF, maximum clot formation), TEG (R, reaction time; K, kinetics; Alpha, alpha angle; MA, maximum amplitude; G-value), and LTA (maximum amplitude, AUC, area under the curve, slope), clopidogrel-induced platelet inhibition (%) was standardized based on the formula:

Percent Inhibition (%) = [(Analyte _Day0_ – Analyte _Day8_)/ Analyte _Day0_] x 100.

For the purpose of this study, cats with percent inhibition <80% on any variables on LTA were considered non- responders (NR) (4).

### 2.7 Statistical analysis

Data analysis was performed using commercially available software (Prism version 10.0a, La Jolla CA, United States). General column statistics were calculated for each method of assessment. Data were tested for normality using a D’Agostino-Pearson omnibus normality test. Parametric data were reported as mean and standard deviation while non-parametric data were reported as median with interquartile range. Normally distributed and paired data were analyzed using paired t-tests while nonparametric and paired data were analyzed using Wilcoxon signed-rank test. Correlation of standardized response to clopidogrel between LTA parameters, VCM Vet and TEG analytes was calculated with Spearman’s rank. Interindividual variability was calculated as the ratio between the standard deviation of a group and its means and expressed as coefficient of variation (CV). Statistical significance was set at *p* < 0.05.

## 3 Results

No adverse event was recorded during the study period. Two cats were excluded from the final analysis due to lipemic serum which resulted in inaccurate measurements of LTA. A total of 28 cats were included in the final analysis.

On Day 0, CBC and biochemistry parameters were all within our laboratory reference ranges. On day 8, platelet count on citrated blood (Day 0: 234.9 × 10^6^/ ml ± 91.6 vs. Day 8: 350.3 × 10^6^/ ml ± 92.4, *p* < 0.0001) and PRP [Day 0: 239 × 10^6^/ ml (169–370.5) vs. Day 8: 401 × 10^6^/ ml (237.5–509), *p* = 0.003] was significantly higher compared to day 0 (Table 1). No other significant difference on CBC parameters was noted on Day 8 compared to Day 0.

**Table 1:** Comparative analysis of CBC and biochemistry variables in 28 cats before (day 0) and after 7 (day 8) days of clopidogrel treatment.

Analyte	Units	Reference interval	Day 0	Day 8	*p*-value
HCT	%	24.0–45.0	43.7 (±4.6)	41.8 (±4.3)	0.076
WBC	x10^9^/L	5.5–19.5	10.2 (±7.8–13.8)	9.8 (8.2–12.6)	0.366
PLT	x10^9^/L	300–800	234.9 (±91.6)	350.0 (±92.4)	**<0.0001**
PLT on PRP	x10^9^/L	N/A	239.0 (169.0–370.5)	401.0 (238.0–509.0)	0.003
TP	g/dL	5.4–8.2	5.2 (±0.4)	N/A	N/A
Alb	g/dL	2.2–4.4	3.2 (±0.3)	N/A	N/A
Glob	g/dL	1.5–5.7	1.9 (±0.3)	N/A	N/A
BUN	mg/dL	10–30	17.4 (±2.6)	N/A	N/A
Crea	mg/dL	0.3–2.1	0.8 (±0.3)	N/A	N/A

HCT, hematocrit; WBC, white blood cell; PLT, platelet; PRP, platelet rich plasma; TP, total protein; Alb, albumin; Glob, globulin; BUN, blood urea nitrogen; Crea, creatinine. Bold value represents statistical significance.

### 3.1 Viscoelastic evaluation (VCM vet and TEG)

**Figure 1:**
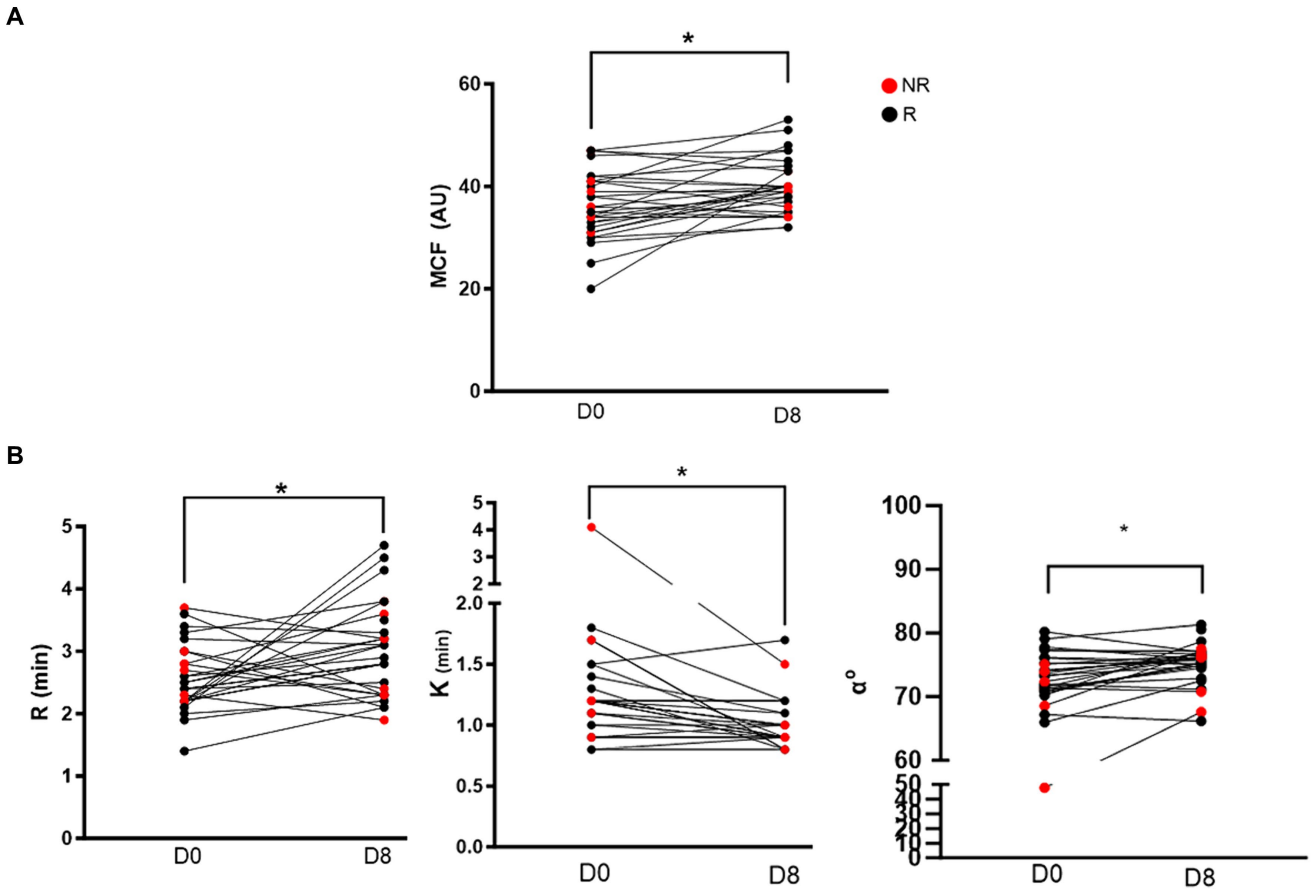
Before-and-after dot plots showing selected viscoelastic variables in 28 cats before (day 0) and after 7 days (day 8) of clopidgrel treatment. Red dots represent non-responders and black dogs represent responders. **(A)** Of the 4 VCM Vet variables, only maximum clot formation (MCF) was significantly increased on day 8 compared to baseline. **(B)** Three of the 5 TEG variables were significantly different after clopidogrel treatment. R-time (min) was prolonged after 7 days of clopdigrol treatment. However, K (min) and alpha angle (*α*°) were significantly decreased and increased, respectively. *p < 0.05.

All VCM Vet and TEG variables are reported in Table 2 with reference ranges generated by our institution reference laboratory (15). VCM Vet analysis demonstrated a significant increase in MCF after 7 days of clopidogrel treatment (Day 0: 36.2 ± 6.5 AU vs. Day 8: 40.1 ± 5.3 AU, *p* = 0.002) (Figure 1A). On TEG, R-time was significantly prolonged after clopidogrel treatment (Day 0: 2.6 min ± 0.5 vs. Day 8: 3.0 min ± 0.7, *p* = 0.024). In contrast, K became shorter [Day 0: 1.2 min (0.9–1.4) vs. 0.9 min (0.8–1.0), *p* = 0.0002], and Alpha angle increased [Day 0: 72.8° (70.8–75.9) vs. 75.9° (74.7–77) 0.0, *p* = 0.0014] after clopidogrel treatment (Figure 1B).

**Table 2:** Comparisons of viscoelastic measurements by VCM Vet, kaolin-activated thromboelastography (TEG), and light transmission aggregometry (LTA) in 28 cats before and after 7 days (Day 0 vs. Day 8) of clopidogrel treatment.

Device	Analyte	Units	Reference interval	Day 0	Day 8	*p*-value
VCM-Vet	CT	Minutes	1.7–6.9	5.8 (±1.65)	7.0 (6.0–7.4)	0.100
	CFT	Minutes	1.7–5.7	3.3 (2.7–3.9)	3.0 (±0.6)	0.196
	AA	Degrees	33.2–66.9	50.5 (43.0–54.0)	48.4 (±6.2)	<0.819
	MCF	VCM Units	22.5–44.8	36.2 (±6.5)	40.1 (±5.3)	**0.002**
TEG	R-time	Minutes	1.7–5.5	2.6 (±0.5)	3.0 (±0.7)	**0.024**
	K	Minutes	0.9–2.1	1.2 (0.9–1.4)	0.9 (0.8–1.0)	**0.0002**
	Alpha	Minutes	61.8–76.8	72.8 (70.8–75.9)	75.9 (74.7–77.0)	**0.001**
	MA	Millimeters	47.1–73.7	58.5 (±6.6)	59.5 (±7.0)	0.619
	G-value	d/scm	4.5–11.2	7.3 (5.7–8.7)	7.5 (5.9–8.9)	0.598
LTA	Max. Amplitude	%	N/A	68.5 (32.2–75.7)	6.0 (3.7–11.7)	**<0.0001**
	Slope	N/A	N/A	94.4 (±30.1)	27.6 (±22.1)	**<0.0001**
	AUC	N/A	N/A	266.5 (132.3–315.3)	18.3 (10.1–36.0)	**<0.0001**

CT, clot time: CFT, clot formation time; AA, alpha angle; MCF, maximum clot formation; R-time, reaction time; MA, maximum amplitude. Bold value represents statistical significance.

### 3.2 ADP-induced light transmission aggregometry

**Figure 2:**
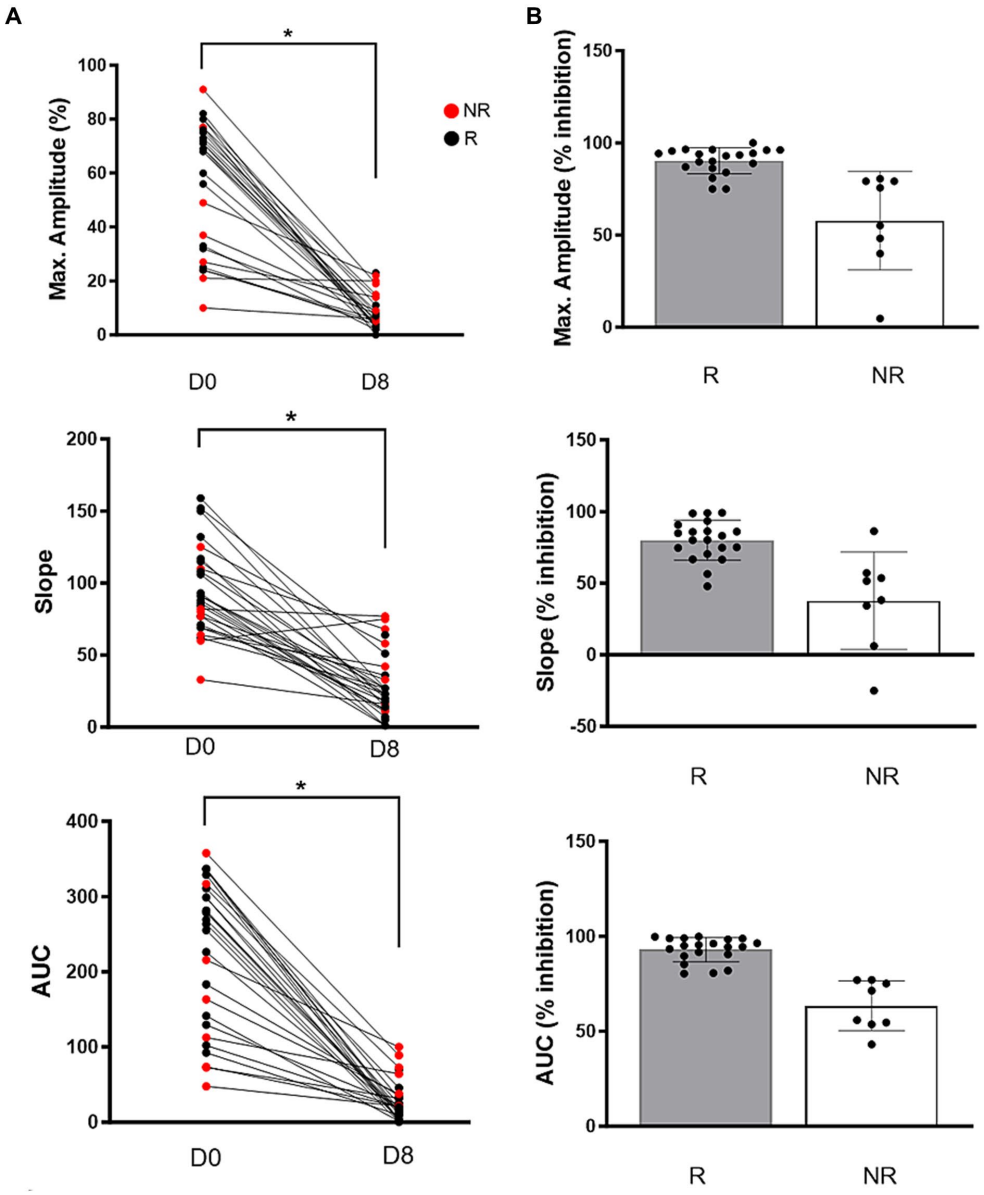
**(A)** Before-and-after dot pots in adenosine diphosphate-induced light transmission aggregometry in 28 cats after 7 days of clopidogrel treatment. Compared to baseline (D0), all variables including maximum amplitude (%), slope and area under the curve (AUC) were significantly decreased following treatment on day 8 (D8). Red dots represent non-responders and black dogs represent responders. **(B)** Clopidogrel response, measured as percent inhibition, is shown on scattered dot pots among responders (R) and non-responders (NR). Bar represents median and error bars show the interquartile ranges *p* < 0.05.

On Day 0, the mean maximum amplitude measured by LTA was 68.5% (32.2–75.7) and decreased significantly after 7 days of clopidogrel treatment [6.0% (3.7–11.7), *p* < 0.0001]. Similarly, AUC [Day 0: 266.5 (132.2–315.3) vs. Day 18.3 (10.1–36.0), p < 0.0001] and slope (Day 0: 94.4 ± 30.1 vs. Day 8: 27.4 ± 22.1 p < 0.0001) were significantly decreased on day 8 (Figure 2A).

### 3.3 Correlations between ADP-induced light-transmission aggregometry and VCM vet, and TEG parameters

We identified a moderate negative correlation between the standardized percentages of inhibition of R-time (TEG) and AUC (LTA) (*R*^2^ = −0.39 *p* = 0.042). No other standardized parameter (TEG and VCM Vet analytes) included in the developed correlation matrix showed a significant correlation with the standardized LTA parameters (Table 3).

**Table 3:** Correlations between clopidogrel-induced platelet inhibition on light transmission aggregometry, VCM Vet and TEG in 28 healthy cats after 7 days of clopidogrel administration.

Device		Light transmission aggregometry		
	Analytes	Amplitude (% inhibition)	Slope (% inhibition)	AUC (% inhibition)
VCM-Vet	CT (% inhibition)	*r* = 0.1 *p* = 0.605	*r* = 0.13 *p* = 0.5	*r* = 0.03 *p* = 0.9
	CFT (% inhibition)	*r* = −0.08 *p* = 0.7	*r* = −0.08 *p* = 0.7	*r* = −0.001 *p* = 1.0
	AA (% inhibition)	*r* = −0.05 *p* = 0.8	*r* = −0.05 *p* = 0.8	*r* = 0.07 *p* = 0.7
	MCF (% inhibition)	*r* = −0.08 *p* = 0.7	*r* = −0.1 *p* = 0.5	*r* = −0.05 *p* = 0.8
TEG	R-time (% inhibition)	*r* = 0.3 *p* = 0.08	*r* = 0.2 *p* = 0.3	***r* = 0.4** ***p* = 0.04**
	K (% inhibition)	*r* = −0.3 *p* = 0.2	*r* = −0.1 *p* = 0.5	*r* = −0.2 *p* = 0.15
	Alpha (% inhibition)	*r* = −0.3 *p* = 0.2	*r* = −0.2 *p* = 0.3	*r* = 0.2 p = 0.3
	MA (% inhibition)	*r* = −0.2 *p* = 0.4	*r* = −0.2 *p* = 0.3	*r* = −0.3 *p* = 0.1
	G-value (% inhibition)	*r* = −0.2 *p* = 0.4	*r* = −0.2 *p* = 0.3	*r* = −0.3 *p* = 0.2

CT, clot time: CFT, clot formation time; AA, alpha angle; MCF, maximum clot formation; R-time, reaction time; MA, maximum amplitude. Bold value represents statistical significance.

Although the overall platelet count was increased in cats after clopidogrel treatment, the degree of platelet increase did not correlate with changes in R (*r* = −0,03, *p* = 0.9), alpha-angle (*r* = −0.06, *p* = 0.75), and K (*r* = −0.03, *p* = 0.90) on TEG. Only MA was significantly and negatively correlated with changes in platelet count (*r* = −0.40, *p* = 0.04). Similarly, of the VCM variables, only change in MCF was significantly correlated with platelet count (*r* = −0.39, *p* = 0.036).

### 3.4 Determining response to clopidogrel

Of the 28 cats, 8 (28.6%) were classified as NRs to clopidogrel based on ADP-induced LTA. Variability in the degree of inhibition was high among NRs (Amplitude: inhibition = 4.8 to 75.8%, CV = 46.1%; Slope: inhibition = −25 to 86.3%, CV = 90.2%; AUC: inhibition = 43.1 to 77%, CV = 20.7%) (Figure 2B). Of the 8 cats, 5 cats (62.5%) had low aggregation (< 40% maximum amplitude) in response to ADP on Day 0 (Figure 2A).

**Figure 3:**
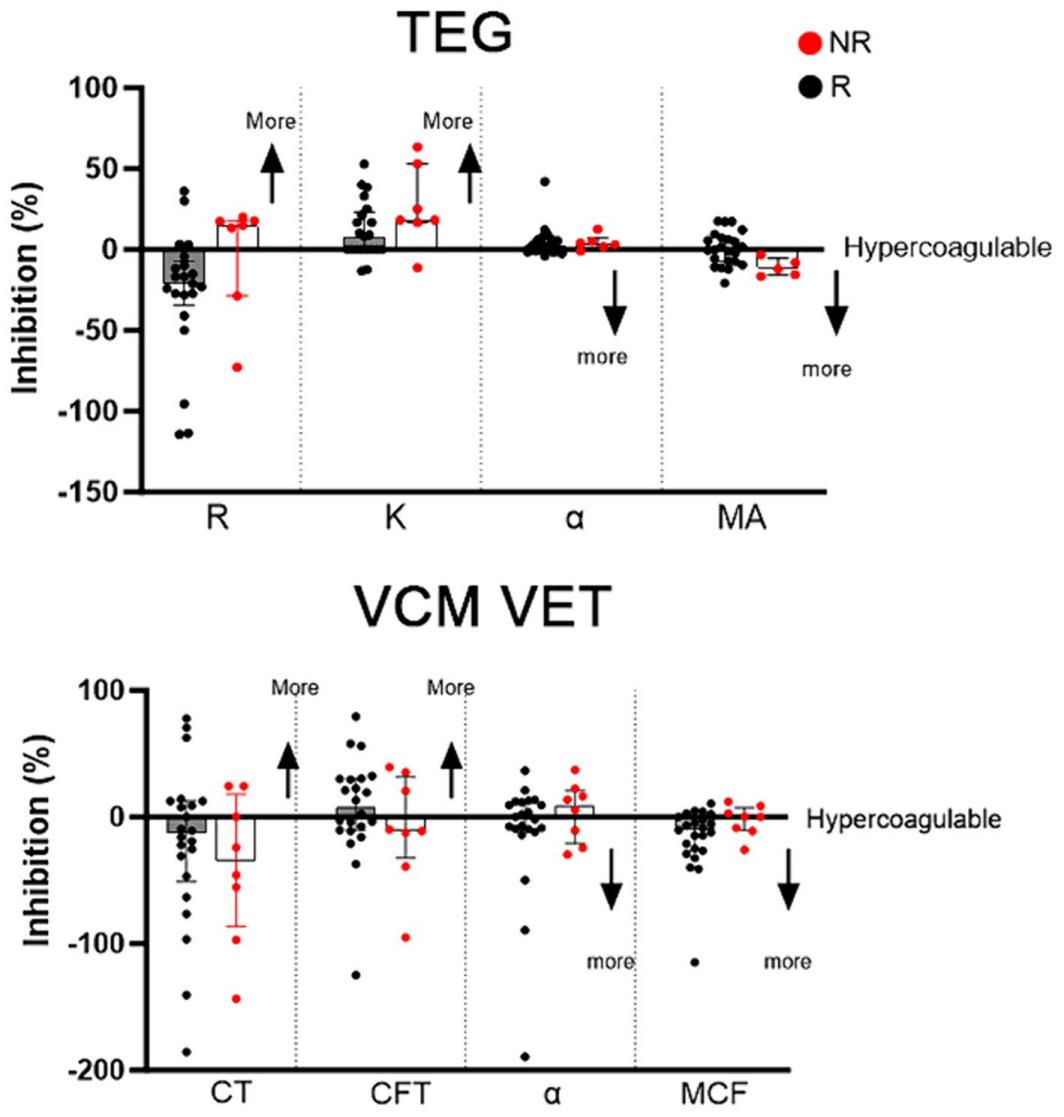
Clopidogrel response, measured as percent inhibition, on kaolin-activated thromboelastography (TEG) and VCM Vet after 7 days of clopdigorel treatment in 28 cats. Red dots represent non-responders and black dogs represent responders. Increased inhibition on R, K, CT and CFT indicates a more hypercoagulable tracing, whereas decreased inhibition on alpha, MA and MCF indicates a more hypercoagulable tracing.

Figure 3 summarizes the changes of TEG and VCM Vet variables among Rs and NRs, identified based on LTA. Of the 8 NRs, 6 (75%) cats had decreased R time on TEG compared to 2 cats in the responder group (10%) (Figure 3A). All NRs (100%) had MA that trended toward more hypercoagulable compared to 10 (50%) responders that had increased MA after clopidogrel treatment. VCM Vet appeared to be less discriminatory in identifying clopidogrel NRs (Figure 3B).

## 4 Discussion

This study aimed to assess the diagnostic utility of two distinct viscoelastic devices in monitoring clopidogrel response in cats. The necessity to perform platelet monitoring following clopidogrel administration is supported by recent research findings in which cats showed a reduced response to clopidogrel, as measured by ADP-induced aggregometry (5). In agreement with previously reported results, in our study population, we identified that 8 out of the 28 cats (28.6%) had decreased response to clopidogrel. Interestingly, a majority of these cats showed a decreased response to ADP prior to clopidogrel treatment and continued to have a modulated response after treatment. One plausible explanation for this finding is that the known non-synonymous single nucleotide polymorphism of the gene, *P2RY*_1_, affects the ADP receptor, *P2Y*_1_, which is not targeted by clopidogrel. This suggests that poor response to ADP may lead to suboptimal levels of inhibition by clopidogrel. This was further confirmed by Ueda et al., who demonstrated that intracellular levels of phosphorylation of vasodilator-stimulated phosphoprotein, which is an indicator of P2Y_12_ inhibition, in cats treated with clopidogrel were not associated with the pharmacogenomic effect of clopidogrel (5). Another explanation is *in vitro* platelet activation, which would compromise platelet response to ADP.

Our research identified that, of all VCM and TEG analytes, only one showed a moderate correlation with the gold standard LTA. According to our analysis, persistent hypercoagulable tracings on kaolin-activated TEG after clopidogrel treatment may identify cats that were poor responders.

Given the high variability in platelet response to ADP, it is, therefore, desirable to identify a platelet function monitoring assay that could be implemented in clinical practice to assess individual response to ADP and clopidogrel therapy. Light transmission aggregometry and flow cytometry have traditionally been considered the gold standard of assessing the efficacy of antiplatelet therapy. These techniques, however, are strictly limited to research settings because they require sophisticated equipment and technical expertise. In recent years, the application of viscoelastic testing has gained popularity in the veterinary field. Until now, TEG has primarily been confined to specialty hospitals and research centers (16). A proprietary modified TEG protocol, TEG Platelet Mapping™ assay (TEG-PM), has been validated in human patients to specifically monitor platelet inhibition following clopidogrel therapy (17). Although a recent publication also demonstrated detectable platelet inhibitory effect in dogs treated with clopidogrel, TEG-PM was found to be unreliable in healthy cats (13, 18). Specifically, when reptilase was used to isolate the contribution of fibrin from platelets to clot formation, increased pre-analytical platelet activation in cats resulted in indistinct differences in MA (13). This finding indicates that TEG-PM using the manufacturer’s protocol may not be able to monitor clopidogrel response in cats. The point-of-care VCM device has recently been introduced to the veterinary market, offering a more readily accessible and widely available viscoelastic testing option (14, 15). In contrast to TEG, VCM analysis is performed on non-anticoagulated blood and does not require any reagents. These factors reduce the technical expertise required but also limit the ability to evaluate the role of individual contributors such as platelets, hematocrit, and fibrin in clot formation.

In our study, only a limited number of variables from the viscoelastic assays showed statistically significant differences after clopidogrel treatment. The prolongation of R on TEG, a measurement of thrombin generation, may be caused by modulation in platelet activation by clopidogrel. During the propagation phase of coagulation, platelets play a central role in facilitating the formation of prothrombinase complex (factors Xa and Va) by externalizing their electronegative phospholipids such as phosphatidylserine (19, 20). This process was previously shown to be further suppressed when cats were treated with dual agent therapy consisting of clopidogrel and rivaroxaban, a direct factor Xa inhibitor (21). An *in vitro* study in humans, however, found that inhibition of platelet integrin did not prolong R on TEG (22). This further highlights the significance of species differences in coagulation. Based on our preliminary findings, 90% of responders were observed to have increased R-time on TEG after clopidogrel treatment compared to a majority of NRs which had decreased R time after treatment. This suggests that R time on TEG may have diagnostic significance in assessing response to clopidogrel. Further studies in clinical cats are needed to verify this finding.

Clot kinetics, while not influenced by platelet count, have been shown to decrease with integrin inhibition in human platelets (22, 23). Notably, some of the observed variables showed a trend toward hypercoagulability after clopidogrel treatment in cats as indicated by an increase in MCF on VCM Vet, increased alpha angle and shortened K on TEG. In TEG, K and alpha angle are measurements of clot kinetics reflecting the rate at which fibrin emerges and polymerizes to form fibrin filaments (24). The underlying mechanism of these unexpected findings is unclear. One possibility is the high inter-assay or operator variability associated with viscoelastic testing and the duration between sampling and testing, which may cause *in vitro* activation of tissue factor or factor XIIa. However, every effort was made to standardize our methods and duration between sampling and assays. Another plausible explanation is that clopidogrel treatment may have prevented *in vitro* activation, allowing platelets to be rested and, thereby, augmenting their response to physiologic agonists during viscoelastic testing. This hyperresponsive nature of feline platelets has previously been shown in cats with increased thrombin generation kinetics in the presence of tissue factor after clopidogrel treatment (21).

After 7 days of clopidogrel treatment, a significant increase in platelet count was observed on CBC. This unexpected finding suggests a potential influence of the administered treatment on platelet dynamics, possibly related to its impact on *in vitro* platelet activation and platelet aggregation. It is plausible that by inhibiting the platelet aggregation process, clopidogrel limits the *in vitro* formation of platelet aggregates, potentially leading to a higher number of platelets detected by automatic counters. However, it is crucial to note that this phenomenon might not necessarily translate to a physiological increase in functional platelets. Careful monitoring is essential to differentiate between the apparent rise in platelet count and any actual changes in platelet physiology or clotting tendencies.

VCM analytes are obtained from non-anticoagulated blood. Without the presence of strong activators like tissue factor or platelet agonists, this assay may not be able to detect changes in platelet function given the variability of the assay and the hyperresponsiveness nature of feline platelets (15). Performing the VCM in series or duplicates could also reduce the inherent variability due to blood draws and platelet clumping. Further studies are needed to investigate if the addition of *in vitro* activators or platelet agonists like ADP in whole blood prior to analysis would augment the ability of the VCM to detect changes in platelet function due to clopidogrel treatment in cats.

The present study has several limitations that should be considered when interpreting the results. First, our study consisted exclusively of healthy colony cats, which may not be entirely representative of the general feline population. The inclusion of only healthy cats may limit the generalizability of our findings to those with underlying health conditions such as cardiomyopathy. In addition, resistance to clopidogrel was not confirmed by genetic testing. Second, the transportation of the blood samples from the cat colony to the central laboratory for analysis is a notable limitation. The handling and transportation of these samples may have introduced variability in sample conditions, which might have caused *in vitro* PLT activation, further confounding the interpretation of our results. An additional limitation of this study is that plasma fibrinogen and coagulation proteins were not measured hence the underlying causes of increased clot kinetics and clot strength on viscoelastic assays could not be determined. Although the collected blood samples were routinely screened for platelet clumping, the detailed information regarding clumping events was not consistently annotated, as the primary focus was on confirming adequate platelet counts in presumably thrombocytopenic samples. This omission restricts our understanding of the full pharmacological effects of clopidogrel in our study population. In light of these limitations, future research should consider a more diverse and representative sample, meticulous sample handling, and the evaluation of active metabolite levels to enhance the comprehensiveness and reliability of the findings.

In conclusion, our study found that while LTA remains the gold standard of monitoring clopidogrel treatment, R-time on TEG has the potential to serve as an alternative monitoring tool. Further clinical studies are needed to confirm these preliminary findings.

## Data availability statement

The original contributions presented in the study are included in the article/Supplementary material, further inquiries can be directed to the corresponding author/s.

## Ethics statement

The animal study was approved by University of California, Davis Institutional Animal Care and Use Committee. The study was conducted in accordance with the local legislation and institutional requirements.

## Author contributions

TR: Conceptualization, Data curation, Formal analysis, Funding acquisition, Investigation, Methodology, Project administration, Resources, Validation, Visualization, Writing – original draft, Writing – review & editing. KJ: Conceptualization, Data curation, Formal analysis, Funding acquisition, Investigation, Methodology, Project administration, Resources, Supervision, Validation, Visualization, Writing – original draft, Writing – review & editing. JS: Conceptualization, Data curation, Funding acquisition, Investigation, Methodology, Project administration, Resources, Supervision, Writing – original draft, Writing – review & editing. NN: Data curation, Formal analysis, Investigation, Methodology, Project administration, Resources, Supervision, Validation, Writing – original draft, Writing – review & editing. RL: Conceptualization, Data curation, Funding acquisition, Investigation, Methodology, Project administration, Resources, Supervision, Writing – original draft, Writing – review & editing, Formal analysis, Validation, Visualization.
